# The impacts of partial replacement of red and processed meat with legumes or cereals on protein and amino acid intakes: a modelling study in the Finnish adult population

**DOI:** 10.1080/07853890.2023.2281661

**Published:** 2023-11-17

**Authors:** Meri Simojoki, Satu Männistö, Heli Tapanainen, Mirkka Maukonen, Liisa M. Valsta, Suvi T. Itkonen, Anne-Maria Pajari, Niina E. Kaartinen

**Affiliations:** a Department of Public Health and Welfare, Finnish Institute for Health and Welfare (THL), Helsinki, Finland; b Department of Food and Nutrition, University of Helsinki, Helsinki, Finland

**Keywords:** amino acids, cereals, legumes, protein, red and processed meat

## Abstract

**Background:**

A shift towards more plant-based diets is considered healthy and environmentally sustainable but may cause a concern regarding protein and amino acid intakes. This modelling study aimed to assess the impacts of partial replacement of red and processed meat with legumes or cereals on the protein and indispensable amino acid intakes in the Finnish adult population.

**Materials and methods:**

We used the cross-sectional data of the National FinDiet 2017 Survey (two non-consecutive 24-h recalls, *n* = 1655, 47% men, aged 18–74 years). Six replacement scenarios were created in which the amount of red and processed meat exceeding 70 g/day (Finnish nutrition recommendation), or 30 g/day (EAT-Lancet recommendation) was replaced with the same amounts of legumes, cereals or their combination. Differences to the reference diet were evaluated based on non-overlapping 95% confidence intervals. Population shares (%) below estimated average requirements (EAR) were calculated using the usual intake modelling methodology (SPADE).

**Results:**

The replacement scenarios decreased the protein and indispensable amino acid intakes depending on gender and age. At the 70-g level, decreases were observed only in men aged 18–64 years. At the 30-g level, decreases were observed in other gender and age groups except women aged 65–74 years. In the scenarios, the mean daily protein intake was 15–18% of total energy intake (E%) (reference 17–18 E%), and the proportions below the EAR were 7–10% in men and 8–10% in women aged 18–64 years (reference 5–7%) and 20–25% in men and 16–20% in women aged 65–74 years (reference 14–17%). For total indispensable amino acids, the proportions below the EAR were <5% in the reference diet and the scenarios.

**Conclusions:**

The mean daily protein intake remained sufficient when red and processed meat was partially replaced with legumes or cereals in the Finnish adult population. However, protein adequacy in the elderly population warrants attention and more research.

## KEY MESSAGES

1. This modelling study supports global efforts towards more plant-based diets, essential to human and planetary health, by providing insight into the impacts of partial replacement of red and processed meat with legumes or cereals on protein and indispensable amino acid intakes in the Finnish adult population.
2. In general, the mean daily protein and indispensable amino acid intakes decreased but remained sufficient also when substantial amounts of red and processed meat were replaced with legumes or cereals.
3. Some elderly men and women, however, had protein intake below the estimated average requirement, which needs to be considered particularly in the transition towards more plant-based and sustainable diets.

## Introduction

Plant-based diets are considered healthy and environmentally sustainable. A shift towards more plant-based diets may reduce the risk of several chronic diseases and mortality [1–3]. Switching towards more plant-based diets also fosters sustainable food production as well as food and nutrition security for the growing world population by promoting the Sustainable Development Goals by United Nations (UN) and the Paris Climate Agreement [4].

From the view of human health and environment, there is a growing need to reduce particularly the consumption of red and processed meat. According to the Nordic nutrition recommendations published in June 2023, the consumption of red and processed meat should be low and not exceed 350 g/week [5]. In the Finnish nutrition recommendations and the World Cancer Research Fund recommendation, the consumption of red and processed meat is limited to less than 500 g/week [6,7]. The EAT-Lancet Commission recently introduced the Planetary Health Diet, which provides a growing world population with healthy diets from sustainable food systems. The Planetary Health Diet is a plant-based diet in which the consumption of red and processed meat is restricted to less than 30 g/day (200 g/week) [4].

The recent review on the food consumption and nutrient intake in the adult population of the Nordic and Baltic countries in 2007–2020 observed that the mean daily intake of meat and meat products varied roughly between 100 and 200 g per day, with higher reported intakes in men than in women [8]. On the other hand, the consumption of pulses, nuts and seeds was very low (pulses 1–18 g/day, nuts and seeds 3–9 g/day). In Finland, the diet of adults has generally improved during 1997–2017 [9]. In men aged 25–64 years, the consumption of red and processed meat decreased between 2012 and 2017. However, 79% of men and 26% of women consume red and processed meat more than 500 g/week [10]. Therefore, a substantial change is needed for the diets to meet these recommendations.

In Western societies, protein covers 15–20% of total energy intake (E%), and two-thirds of protein is derived from animal sources and one-third from plant sources [10–14]. Meat and other animal-based foods are rich in high-quality protein [5]. Plant-based foods, such as legumes and cereals, also provide protein, but in lower amounts, and the proportions of indispensable amino acids are not as optimal as in animal-based foods. The digestibility of protein is also lower in plant-based foods than in animal-based foods [15,16]. A shift from animal-based diets to more plant-based diets might therefore result in somewhat lower protein and amino acid intakes. In plant-based diets, a variety of foods providing plant proteins is essential. For instance, a combination of plant proteins from legumes and cereals will give a good distribution of indispensable amino acids [5].

The Nordic and Finnish nutrition recommendations for protein intake are 10–20 E% for adults aged 18–64 years and 15–20 E% for adults aged 65 years or more [5,6]. For most of the Finnish adult population, the intake of protein is according to the recommendations [10]. However, in 19% of men and 15% of women aged 65–74 years protein intake is below the recommendation. Therefore, a shift towards more plant-based diets may cause a concern regarding protein and indispensable amino acid intakes, particularly in vulnerable groups such as the elderly. Optimising protein intake in elderly populations is important to prevent age-related loss of muscle mass and decline of physical functioning [5,17].

Intervention studies regarding the impacts of animal-based diets versus plant-based diets on protein intake are scarce. In a 12-week randomised clinical trial in healthy 20–69-year-old Finnish adults, animal protein sources were partially replaced with plant-based ones [18–20]. The mean protein intake tended to be lower in more plant-based diets but still, on average, provided the recommended intake for this working-age population. Thus far, a few modelling studies have assessed the impacts of the replacement of animal-based foods with plant-based foods on nutrient intakes in large population-based samples. These studies have been conducted in US populations aged ≥2 years [21] and ≥51 years [22], in the Canadian population aged ≥1 year [23], in the Dutch population aged 19–69 years [24], in the French population aged 18–79 years [25–27], in the Swedish population aged 18–80 years [28] and in the Finnish population aged 18–74 years [29]. According to previous studies, a transition towards more plant-based diets tended to decrease the protein intake and increase the proportion of the population not meeting the estimated average requirement (EAR) for protein intake. However, in only two of these studies, protein intakes in older age groups were studied [22,24]. Also, in only two of these studies, the intakes of specific amino acids were analysed [26, 27]. Therefore, more research is needed on the impact of various plant-based diets on protein and amino acid intakes in specific population groups.

This theoretical modelling study aimed to assess the impacts of partial replacement of red and processed meat with legumes or cereals on the intakes of protein and indispensable amino acids in the different gender and age groups of the Finnish adult population. In addition, changes in population shares of protein and indispensable amino acid intakes below EAR were explored. Our hypothesis is that the mean protein and indispensable amino acid intakes in the Finnish adult population remain sufficient when red and processed meat is partially replaced with legumes or cereals. These data are needed to support nutritional policy efforts to secure adequate nutrition for all population groups in transition towards more plant-based and sustainable diets.

## Materials and methods

### Study population

We used the cross-sectional data of the National FinDiet 2017 Survey [10,30]. The data were collected as part of the National FinHealth 2017 Study which included health examination and aimed producing reliable information on health, wellbeing and functional capacity in the Finnish adult population [31, 32]. For the FinHealth 2017 Study, an eligible sample of adults aged 18 years and above was drawn from the Population Register (*n* = 10,247) using stratified one- and two-staged sampling design. In all, 30% (*n* = 3099) were invited to the FinDiet 2017 Survey. Of them, 59% participated in the health examination and were eligible to the dietary data collection (i.e. two non-consecutive 24-h dietary recalls). At the study clinic, 29 subjects refused or were not able to participate in the first dietary recall, 114 subjects were not reached for the second dietary recall, and 16 subjects were excluded due to one or two incomplete dietary recalls. The final FinDiet 2017 Survey data consisted of two non-consecutive 24-h recalls from 875 women and 780 men aged 18–74 years (altogether 1655 participants, 53% of the original sub-sample). Non-participation bias was corrected using weighing factors, which improve the representativeness of the results to the Finnish adult population [33].

### Ethical considerations

This study was conducted according to the guidelines laid down in the Declaration of Helsinki, and all procedures involving research study participants were approved by the Coordinating Ethics Committee at the Hospital District of Helsinki and Uusimaa (Reference 37/13/03/00/2016). Written informed consent was obtained from all subjects.

### Background characteristics

For this study, subject’s gender and age were obtained from the sampling frame. Weight and height were measured during health examination by trained research staff according to standard protocols [34].

### Dietary assessment

Two non-consecutive 24-h dietary recalls were conducted by 10 uniformly trained dietary interviewers according to EU Menu methodology [30]. The first was conducted as an in-person interview in the health examination in January–May 2017, and the second was conducted as a telephone interview in February–October 2017. The two 24-h dietary recall interviews took place at a minimum of 8 days apart. All four seasons and all days of the week were covered in the data collection.

### Food consumption and nutrient intake

The food consumption and nutrient intake were calculated using the in-house calculation software Finessi and the Finnish national food composition database Fineli® [35,36]. The daily consumption of red and processed meat (g), legumes (g) and cereals (g) and the daily intakes of energy (MJ), total fat (E%), saturated fat (E%), monounsaturated fat (E%), carbohydrate (E%), fibre (g/MJ), protein (E%, g, g/kg body weight) and indispensable amino acids (mg/kg body weight) were calculated. Also, the proportion of animal protein and plant protein in total protein intake were evaluated. Sulphur amino acids methionine and cysteine were summed up, as well as aromatic amino acids phenylalanine and tyrosine [15].

The consumption of red and processed meat included the consumption of beef, pork, lamb, game, and offal, all as cooked and meat products such as sausages and cold cuts. The consumption of legumes included all forms of fresh legumes (green peas or green beans), pulses (dry beans, chickpeas, lentils), soya products and legume-based plant-protein products. The consumption of cereals included barley, oat, rice, rye, wheat, starch, other cereals and cereal-based plant-protein products excluding cereal-based drinks.

The amino acid composition data of 236 key protein sources were recently updated for Fineli® [37,38] and used in these calculations. The updated amino acid composition data were derived from Finnish publications, Frida food composition database from Denmark and USDA food composition database from the United States.

For the purposes of this study, legume, cereal and a combination aggregate were created based on the data of the National FinDiet 2017 Survey [10]. Most-commonly consumed legume and cereal foods according to the FinDiet 2017 Survey were identified, and their relative contribution to the legume and cereal aggregates were calculated based on user frequencies. The legume aggregate consisted of cooked legumes: green peas (45%), soya mince (10%), green bean and string bean (10%), kidney bean (10%), white bean (5%), chickpea (5%), red lentils (5%), faba bean and pea protein product Härkis® (5%) and pulled oats (5%) [29]. The cereal aggregate consisted of rye bread (59%), oatmeal porridge (13%), oatmeal bread (10%), wheat bread (7%), cooked rice (6%) and cooked pasta (5%). The combination aggregate consisted of legume aggregate (50%) and cereal aggregate (50%). The legume, cereal and combination aggregate and their nutrient contents were used in the replacement scenario analyses (see below). The energy and nutrient composition per 100 g of the aggregates are presented in Table S1.

### Statistical analysis

Six replacement scenarios were created in this study. In the scenarios, the amount of red and processed meat that exceeded 70 g/day (corresponding to the Finnish nutrition recommendation maximum 500 g/week [6]) or 30 g/day (corresponding to the EAT-Lancet recommendation maximum 200 g/week [4]) was replaced with the same amount (g) of legume, cereal, or the combination aggregate (see above) while keeping the consumption of other foods unchanged.

In the replacement scenarios, cooked red meat was replaced with cooked legumes or cereals. An average conversion factor of 0.7 was applied to convert the ingredient consumption of red meat into cooked red meat consumption (500 g cooked red meat corresponds to 700–750 g uncooked meat). For processed meat, no conversion factor was used.

Mean daily food consumption and nutrient intake and their 95% confidence intervals were calculated for the current (reference) diet and the replacement scenarios by gender and age groups (18–64, 65–74 years). The mean and 95% confidence intervals were calculated by means of deterministic approach taking into account survey weights and the sampling frame. In the reference diet, significant differences in background characteristics, food consumption and nutrient intakes between gender and age groups were tested using linear regression. In the linear regression analyses, the response variables were log or cube root transformed before the analyses, when necessary, to achieve normality. For legumes, due to low proportion of legume users (<50%), logistic regression was used to test the differences between the groups. For the replacement scenarios, standard statistical testing was not appropriate as the food consumption data had been altered by the study design. Instead, meaningful differences between the reference diet and the scenarios were examined using non-overlapping 95% confidence intervals as in previous studies [21,39].

The proportions of the population not meeting the EAR for the protein and indispensable amino acid intakes were assessed for the reference diet, the legume scenarios and the cereal scenarios, using the SPADE method that models usual intake distributions using short-term measurements (Statistical Program to Assess Dietary Exposure, RIVM, the Netherlands) [40]. In the combination scenarios, proportions were not calculated because estimates were assumed to fall between the legume and cereal scenarios. SPADE uses a probabilistic approach and models intake of a dietary component as a function of age. Meaningful differences between the reference diet and the scenarios were not examined because the changes in protein and indispensable amino acid intakes were relatively small. The EAR values used in this study were those estimated by the WHO/FAO/UNU [15].

## Results

### Food consumption and nutrient intakes in the reference diet

In the reference diet, the mean daily consumption of red and processed meat was higher in men aged 18–64 years (119 g) compared to men aged 65–74 years (97 g) (Table 1). In women, the mean daily consumption of red and processed meat was half that of men, 59 g in women aged 18–64 years and 54 g in women aged 65–74 years. The proportion of legume users was higher in men and women aged 18–64 years compared to men and women aged 65–74 years. The mean daily consumption of cereals was higher in working age compared to older men and women and in men compared to women in both age groups.

**Table 1.** Background characteristics, mean daily food consumption and nutrient intake in the FinDiet 2017 Survey by gender and age.

	Men (*n* = 780)			Women (*n* = 875)		
	18–64 years (*n* = 576)	65–74 years (*n* = 204)		18–64 years (*n* = 628)	65–74 years (*n* = 247)	
	Mean	Mean	*P* value^c^	Mean	Mean	*P* value^c^
Age (years)	42.1	69.2	–	41.5	69.2	–
Body weight (kg)	86.1^a^	88.0^a^	NS	72.6	74.9	NS
Body mass index (kg/m^2^)	27.1	28.7	<0.0001	26.9	28.7	<0.0001
Red and processed meat (g)	119^a^	96.6^a^	0.020	59.3	54.4	NS
Legumes (g)	13.4	7.1	0.027^b^	14.0	9.4	<0.0001^b^
Cereals (g)	152^a^	138^a^	0.013	113	104	0.036
Energy (MJ)	9.8^a^	8.0^a^	<0.0001	7.5	6.6	<0.0001
Fat (E%)	39.0^a^	36.7	<0.0001	38.0	36.7	NS
Saturated fat (E%)	15.2^a^	14.4	0.017	14.4	14.0	NS
Monounsaturated fat (E%)	14.8	13.4	<0.0001	14.5	13.7	0.0059
Carbohydrate (E%)	40.8^a^	44.0	<0.0001	42.2	43.4	NS
Fibre (g/MJ)	2.4^a^	2.9^a^	<0.0001	2.8	3.1	<0.0001
Protein (E%)	18.2^a^	17.0	<0.0001	17.5	17.3	NS
Animal source (% of total protein)	70.1^a^	66.5	<0.0001	67.4	67.1	NS
Plant source (% of total protein)	29.8^a^	33.3	<0.0001	32.3	32.8	NS
Protein (g/kg body weight (BW))	1.2^a^	0.89	<0.0001	1.1	0.91	<0.0001
Histidine (mg/kg BW)	37.3^a^	26.9	<0.0001	32.4	27.4	<0.0001
Isoleucine (mg/kg BW)	50.7^a^	35.7	<0.0001	44.7	37.4	<0.0001
Leucine (mg/kg BW)	93.7^a^	66.2	<0.0001	82.9	69.4	<0.0001
Lysine (mg/kg BW)	82.3^a^	57.7	<0.0001	71.4	60.2	<0.0001
Methionine + cysteine (mg/kg BW)	43.1^a^	31.4	<0.0001	38.3	32.3	<0.0001
Phenylalanine + tyrosine (mg/kg BW)	95.2^a^	68.1	<0.0001	85.5	71.8	<0.0001
Threonine (mg/kg BW)	44.3^a^	31.5	<0.0001	39.2	33.0	<0.0001
Tryptophan (mg/kg BW)	15.9^a^	11.5	<0.0001	14.3	12.0	<0.0001
Valine (mg/kg BW)	60.0^a^	42.8	<0.0001	53.7	45.1	<0.0001
Total indispensable amino acids (mg/kg BW)	522^a^	372	<0.0001	463	389	<0.0001

^c^Difference between age groups tested by linear regression.

NS, not significant.

^a^Different from women of the same age group (*p* < 0.05), tested by linear regression.

^b^Due to low proportion of legume users (<50%), difference in proportion of users between age and gender groups tested by logistic regression. No differences between men and women of the same age group were observed (*p* > 0.05).

In men aged 18–64 years, the mean daily intake of energy (MJ), total fat (E%), saturated fat (E%), monounsaturated fat (E%), protein (E%, g/kg body weight (BW)) and indispensable amino acids (mg/kg BW) were higher, and carbohydrate (E%) and fibre (g/MJ) lower compared to men aged 65–74 years (Table 1). Also, men of working age had a higher proportion of animal protein (70%) and a lower proportion of plant protein (30%) in total protein intake compared to older men (67% and 33%, respectively). In women aged 18–64 years, the mean daily intake of energy (MJ), monounsaturated fat (E%), protein (g/kg BW) and indispensable amino acids (mg/kg BW) were higher and fibre (g/MJ) was lower compared to women aged 65–74 years.

Gender differences in nutrient intakes were particularly observed in subjects aged 18–64 years (Table 1). In men aged 18–64 years, the mean daily intake of energy (MJ), total fat (E%), saturated fat (E%), protein (E%, g/kg BW) and indispensable amino acids (mg/kg BW) were higher, and carbohydrate (E%) and fibre (g/MJ) lower compared to women aged 18–64 years. Also, men of working age had a higher proportion of animal protein and a lower proportion of plant protein in their diet compared to women of the same age. In men aged 65–74 years, the mean daily intake of energy (MJ) was higher, and fibre (g/MJ) was lower compared to women aged 65–74 years.

The mean daily energy intake ranged from 6.6 MJ in older women to 9.8 MJ in men of working age. The mean daily protein intake ranged from 17 E% (0.89 g/kg BW) in older men to 18 E% (1.2 g/kg BW) in men of working age.

### Replaced quantities of red and processed meat

The consumption of red and processed meat exceeded 70 g/day in 68% of men aged 18-64 years and 57% of men aged 65–74 years (data not shown). In women, the corresponding proportions were 36% and 27%, respectively. The consumption of red and processed meat exceeded 30 g/day in 86% of men aged 18–64 and 65–74 years, whereas in women the corresponding proportions were 69% and 63%, respectively. The average daily quantities of red and processed meat to be replaced by legumes or cereals were 80 g/day in men and 42 g/day in women at the 70-g level. At the 30-g level, the corresponding quantities were 96 g/day in men and 51 g/day in women.

### Protein and indispensable amino acid intakes in the replacement scenarios

The replacement scenarios decreased the protein and indispensable amino acid intakes depending on gender and age (Tables 2–5). Decreases in the protein and indispensable amino acid intakes were particularly observed in men aged 18–64 years (Table 2).

**Table 2.** Mean daily food consumption and nutrient intake in Finnish men (*n* = 576) aged 18–64 years in the reference diet (FinDiet 2017) and the scenarios in which red and processed meat were partially replaced with legumes or cereals^a^.

	Level	Reference diet		Legume scenario		Cereal scenario		Combination scenario	
		Mean	95% CI	Mean	95% CI	Mean	95% CI	Mean	95% CI
Red and processed meat (g)^b^	70	119	111–127	58.9^c^	56.9–61.0	58.9^c^	56.9–61.0	58.9^c^	56.9–61.0
	30			27.7^c^	27.0–28.4	27.7^c^	27.0–28.4	27.7^c^	27.0–28.4
Legumes (g)^d^	70	13.4	9.6–17.1	69.3^c^	61.2–77.5	13.4	9.6–17.1	41.3^c^	35.9–46.8
	30			98.6^c^	89.8–107	13.4	9.6–17.1	56.0^c^	50.3–61.7
Cereals (g)^e^	70	152	144–161	152	144–161	186^c^	176–196	169	160–178
	30			152	144–161	203^c^	193–213	178^c^	169–187
Energy (MJ)	70	9.8	9.5–10.1	9.5	9.1–9.8	9.7	9.4–10.0	9.6	9.3–9.9
	30			9.3	9.0–9.6	9.7	9.4–10.0	9.5	9.2–9.8
Protein (E%)	70	18.2	17.9–18.6	17.7	17.4–18.1	17.0^c^	16.7–17.4	17.4^c^	17.0–17.7
	30			17.4^c^	17.0–17.7	16.3^c^	16.0–16.7	16.8^c^	16.5–17.2
Protein (g)	70	102	98.3–107	95.9	91.9–99.8	94.8	90.9–98.7	95.3	91.4–99.2
	30			92.2^c^	88.4–96.1	90.6^c^	86.8–94.4	91.4^c^	87.6–95.3
Protein (g/kg BW)	70	1.2	1.2–1.3	1.1	1.1–1.2	1.1	1.1–1.2	1.1	1.1–1.2
	30			1.1^c^	1.05–1.15	1.1^c^	1.0–1.1	1.1^c^	1.0–1.1
Histidine (mg/kg BW)	70	37.3	35.7–38.9	33.5^c^	32.1–34.9	33.1^c^	31.7–34.5	33.3^c^	31.9–34.7
	30			31.4^c^	30.0–32.7	30.8^c^	29.5–32.2	31.1^c^	29.7–32.4
Isoleucine (mg/kg BW)	70	50.7	48.4–52.9	47.2	45.1–49.4	46.3	44.2–48.5	46.8	44.6–48.9
	30			45.3^c^	43.2–47.4	44.0^c^	41.8–46.1	44.6^c^	42.5–46.7
Leucine (mg/kg BW)	70	93.7	89.5–97.9	87.6	83.7–91.6	86.4	82.5–90.4	87.0	83.1–91.0
	30			84.2^c^	80.3–88.1	82.4^c^	78.5–86.3	83.3^c^	79.4–87.2
Lysine (mg/kg BW)	70	82.3	78.6–86.1	74.8^c^	71.4–78.3	74.1^c^	70.7–77.6	74.5^c^	71.1–77.9
	30			70.7^c^	67.3–74.0	69.6^c^	66.3–72.9	70.1^c^	66.8–73.5
Methionine + cysteine (mg/kg BW)	70	43.1	41.2–45.0	39.3^c^	37.5–41.0	39.8	38.0–41.5	39.5	37.7–41.3
	30			37.1^c^	35.4–38.9	37.9^c^	36.2–39.6	37.5^c^	35.8–39.2
Phenylalanine + tyrosine (mg/kg BW)	70	95.2	91.1–99.4	90.2	86.3–94.1	89.7	85.8–93.7	90.0	86.0–93.9
	30			87.4	83.5–91.3	86.7^c^	82.8–90.5	87.0^c^	83.2–90.9
Threonine (mg/kg BW)	70	44.3	42.3–46.3	41.3	39.4–43.2	40.4	38.6–42.3	40.9	39.0–42.8
	30			39.7^c^	37.8–41.5	38.3^c^	36.5–40.1	39.0^c^	37.1–40.8
Tryptophan (mg/kg BW)	70	15.9	15.2–16.6	14.8	14.2–15.5	14.7	14.1–15.4	14.8	14.1–15.4
	30			14.2^c^	13.6–14.9	14.0^c^	13.4–14.7	14.1^c^	13.5–14.8
Valine (mg/kg BW)	70	60.0	57.4–62.6	56.4	54.0–58.9	55.8	53.4–58.3	56.1	53.7–58.6
	30			54.5^c^	52.0–56.9	53.5^c^	51.1–56.0	54.0^c^	51.6–56.4
Total indispensable amino acids (mg/kg BW)	70	522	499–546	485	464–507	480	459–502	483	461–504
	30			464^c^	443–486	457^c^	436–478	461^c^	440–482

^a^In the replacement scenarios, the amount of red and processed meat exceeding 70 g/day or 30 g/day was replaced with the same amount (g) of legume, cereal or the combination (legume 50%, cereal 50%) aggregate while keeping the consumption of other foods unchanged.

^b^Red meat included beef, pork, lamb, game and offal and processed meat included sausages and cold cuts.

^c^Different from the reference diet based on non-overlapping 95% confidence interval (CI) around the mean [21,39].

^d^Legumes included all forms fresh legumes (green peas or green beans), pulses (dry beans, chickpeas, lentils), soya products and legume-based plant-protein products.

^e^Cereals included barley, oat, rice, rye, wheat, starch, other cereals and cereal-based plant-protein products excluding cereal-based drinks.

In men aged 18–64 years, the legume scenario decreased the intakes of histidine, lysine and methionine + cysteine, whereas the cereal scenario and the combination scenario decreased the intakes of protein (E%), histidine and lysine at the 70-g level (Table 2). At the 30-g level, the replacement scenarios decreased the intakes of protein (E%, g, g/kg BW) and all indispensable amino acids except phenylalanine + tyrosine in the legume scenario.

In other gender and age groups, no meaningful differences were observed in the protein and indispensable amino acid intakes between the reference diet and the scenarios at the 70-g level (Tables 3–5). At the 30-g level, decreases compared to the reference diet were observed in men aged 65–74 years and women aged 18–64 years but not in women aged 65–74 years.

**Table 3.** Mean daily food consumption and nutrient intake in Finnish men (*n* = 204) aged 65–74 years in the reference diet (FinDiet 2017) and the scenarios in which red and processed meat were partially replaced with legumes or cereals^a^.

	Level	Reference diet		Legume scenario		Cereal scenario		Combination scenario	
		Mean	95% CI	Mean	95% CI	Mean	95% CI	Mean	95% CI
Red and processed meat (g)^b^	70	96.6	84.3–109	57.3^c^	54.0–60.6	57.3^c^	54.0–60.6	57.3^c^	54.0–60.6
	30			28.3^c^	27.3–29.2	28.3^c^	27.3–29.2	28.3^c^	27.3–29.2
Legumes (g)^d^	70	7.1	4.9–9.4	44.0^c^	33.4–54.6	7.1	4.9–9.4	25.6^c^	19.8–31.3
	30			71.3^c^	59.5–83.0	7.1	4.9–9.4	39.2^c^	32.9–45.5
Cereals (g)^e^	70	138	130–146	138	130–146	160^c^	150–170	149	141–158
	30			138	130–146	176^c^	165–187	157^c^	148–166
Energy (MJ)	70	8.0	7.6–8.3	7.8	7.5–8.1	8.0	7.6–8.3	7.9	7.6–8.2
	30			7.7	7.3–8.0	8.0	7.6–8.3	7.8	7.5–8.1
Protein (E%)	70	17.0	16.4–17.5	16.6	16.1–17.1	16.1	15.6–16.6	16.4	15.9–16.8
	30			16.2	15.8–16.7	15.3^c^	14.8–15.8	15.8^c^	15.3–16.2
Protein (g)	70	76.1	72.6–79.6	72.6	69.5–75.7	71.9	68.9–75.0	72.3	69.2–75.4
	30			69.7	66.7–72.7	68.4^c^	65.5–71.3	69.0^c^	66.1–72.0
Protein (g/kg BW)	70	0.89	0.84–0.94	0.85	0.81–0.89	0.84	0.80–0.88	0.85	0.80–0.89
	30			0.82	0.77–0.86	0.80^c^	0.76–0.84	0.81	0.77–0.85
Histidine (mg/kg BW)	70	26.9	25.4–28.5	24.7	23.5–26.0	24.5	23.3–25.7	24.6	23.4–25.8
	30			22.9^c^	21.8–24.0	22.5^c^	21.4–23.6	22.7^c^	21.6–23.8
Isoleucine (mg/kg BW)	70	35.7	33.8–37.6	33.8	32.1–35.5	33.2	31.6–34.8	33.5	31.9–35.2
	30			32.2^c^	30.6–33.8	31.2^c^	29.6–32.7	31.7^c^	30.1–33.2
Leucine (mg/kg BW)	70	66.2	62.7–69.7	62.8	59.7–65.9	62.0	59.0–65.0	62.4	59.3–65.4
	30			59.9	56.9–62.9	58.5^c^	55.6–61.4	59.2^c^	56.3–62.2
Lysine (mg/kg BW)	70	57.7	54.4–61.0	53.4	50.7–56.2	53.0	50.3–55.7	53.2	50.5–55.9
	30			49.9^c^	47.3–52.5	49.1^c^	46.5–51.6	49.5^c^	46.9–52.1
Methionine + cysteine (mg/kg BW)	70	31.4	29.7–33.1	29.2	27.7–30.6	29.5	28.0–31.0	29.3	27.9–30.8
	30			27.4^c^	26.0–28.7	27.9^c^	26.5–29.3	27.6^c^	26.3–29.0
Phenylalanine + tyrosine (mg/kg BW)	70	68.1	64.7–71.6	65.5	62.2–68.7	65.1	62.0–68.3	65.3	62.1–68.5
	30			63.1	60.0–66.3	62.6	59.5–65.7	62.8	59.7–66.0
Threonine (mg/kg BW)	70	31.5	29.7–33.2	29.8	28.3–31.3	29.2	27.7–30.7	29.5	28.0–31.0
	30			28.4	26.9–29.8	27.4^c^	26.0–28.7	27.9^c^	26.4–29.3
Tryptophan (mg/kg BW)	70	11.5	10.9–12.1	10.9	10.3–11.4	10.8	10.3–11.3	10.8	10.3–11.4
	30			10.4	9.8–10.9	10.2^c^	9.7–10.7	10.3^c^	9.8–10.8
Valine (mg/kg BW)	70	42.8	40.6–45.0	40.9	38.9–42.9	40.5	38.5–42.4	40.7	38.7–42.7
	30			39.2	37.3–41.2	38.5^c^	36.6–40.4	38.9	36.9–40.8
Total indispensable amino acids (mg/kg BW)	70	372	352–392	351	334–368	348	331–365	349	332–367
	30			333^c^	317–350	328^c^	312–344	331^c^	314-347

^a^In the replacement scenarios, the amount of red and processed meat exceeding 70 g/day or 30 g/day was replaced with the same amount (g) of legume, cereal or the combination (legume 50%, cereal 50%) aggregate while keeping the consumption of other foods unchanged.

^b^Red meat included beef, pork, lamb, game and offal and processed meat included sausages and cold cuts.

^c^Different from the reference diet based on non-overlapping 95% confidence interval (CI) around the mean [21,39].

^d^Legumes included all forms fresh legumes (green peas or green beans), pulses (dry beans, chickpeas, lentils), soya products and legume-based plant-protein products.

^e^Cereals included barley, oat, rice, rye, wheat, starch, other cereals and cereal-based plant-protein products excluding cereal-based drinks.

**Table 4.** Mean daily food consumption and nutrient intake in Finnish women (*n* = 628) aged 18–64 years in the reference diet (FinDiet 2017) and the scenarios in which red and processed meat were partially replaced with legumes or cereals^a^.

	Level	Reference diet		Legume scenario		Cereal scenario		Combination scenario	
		Mean	95% CI	Mean	95% CI	Mean	95% CI	Mean	95% CI
Red and processed meat (g)^b^	70	59.3	55.0–63.6	44.1^c^	41.7–46.6	44.1^c^	41.7–46.6	44.1^c^	41.7–46.6
	30			23.8^c^	22.7–24.8	23.8^c^	22.7–24.8	23.8^c^	22.7–24.8
Legumes (g)^d^	70	14.0	10.9–17.2	28.2^c^	25.1–31.4	14.0	10.9–17.2	21.1^c^	18.2–24.1
	30			47.3^c^	43.9–50.8	14.0	10.9–17.2	30.7^c^	27.9–33.5
Cereals (g)^e^	70	113	108–117	113	108–117	121	117–126	117	113–122
	30			113	108–117	133^c^	128–138	123^c^	118–128
Energy (MJ)	70	7.5	7.4–7.7	7.5	7.3–7.6	7.5	7.3–7.7	7.5	7.3–7.7
	30			7.3	7.2–7.5	7.5	7.3–7.7	7.4	7.2–7.6
Protein (E%)	70	17.5	17.2–17.8	17.3	17.0–17.6	17.1	16.8–17.4	17.2	16.9–17.5
	30			17.0	16.7–17.3	16.5^c^	16.2–16.8	16.8^c^	16.5–17.1
Protein (g)	70	75.2	73.3–77.2	73.7	71.8–75.5	73.4	71.6–75.3	73.6	71.7–75.4
	30			71.3^c^	69.5–73.1	70.7^c^	68.9–72.5	71.0^c^	69.2–72.8
Protein (g/kg BW)	70	1.1	1.0–1.1	1.1	1.0–1.1	1.1	1.0–1.1	1.1	1.0–1.1
	30			1.0	0.99–1.1	1.0	0.98–1.1	1.0	0.99–1.1
Histidine (mg/kg BW)	70	32.4	31.2–33.6	31.3	30.2–32.4	31.2	30.1–32.3	31.2	30.1–32.4
	30			29.6^c^	28.5–30.7	29.4^c^	28.3–30.4	29.5^c^	28.4–30.6
Isoleucine (mg/kg BW)	70	44.7	43.1–46.3	43.8	42.2–45.3	43.5	41.9–45.0	43.6	42.1–45.2
	30			42.3	40.7–43.8	41.6	40.1–43.1	41.9	40.4–43.4
Leucine (mg/kg BW)	70	82.9	80.0–85.9	81.2	78.4–84.1	80.8	78.0–83.7	81.0	78.2–83.9
	30			78.6	75.8–81.3	77.7	74.9–80.5	78.1	75.4–80.9
Lysine (mg/kg BW)	70	71.4	68.8–74.0	69.3	66.7–71.8	69.0	66.5–71.6	69.2	66.6–71.7
	30			66.0^c^	63.6–68.4	65.5^c^	63.1–67.9	65.8^c^	63.3–68.2
Methionine + cysteine (mg/kg BW)	70	38.3	36.9–39.6	37.1	35.9–38.4	37.3	36.0–38.6	37.2	35.9–38.5
	30			35.5^c^	34.3–36.7	35.9	34.6–37.1	35.7^c^	34.4–36.9
Phenylalanine + tyrosine (mg/kg BW)	70	85.5	82.5–88.5	84.1	81.2–87.0	84.0	81.0–86.9	84.0	81.1–87.0
	30			81.9	79.1–84.8	81.6	78.7–84.4	81.8	78.9–84.6
Threonine (mg/kg BW)	70	39.2	37.8–40.6	38.4	37.0–39.8	38.1	36.8–39.5	38.3	36.9–39.6
	30			37.1	35.8–38.4	36.5^c^	35.2–37.8	36.8	35.5–38.1
Tryptophan (mg/kg BW)	70	14.3	13.8–14.8	14.0	13.5–14.5	14.0	13.5–14.5	14.0	13.5–14.5
	30			13.5	13.1–14.0	13.5	13.0–13.9	13.5	13.0–14.0
Valine (mg/kg BW)	70	53.7	51.8–55.6	52.7	50.9–54.6	52.5	50.7–54.4	52.6	50.8–54.5
	30			51.2	49.4–53.0	50.8	49.0–52.5	51.0	49.2–52.8
Total indispensable amino acids (mg/kg BW)	70	463	446–479	452	436–468	450	435–466	451	435–467
	30			436	420–451	432	417–448	434	419–449

^a^In the replacement scenarios, the amount of red and processed meat exceeding 70 g/day or 30 g/day was replaced with the same amount (g) of legume, cereal or the combination (legume 50%, cereal 50%) aggregate while keeping the consumption of other foods unchanged.

^b^Red meat included beef, pork, lamb, game and offal and processed meat included sausages and cold cuts.

^c^Different from the reference diet based on non-overlapping 95% confidence interval (CI) around the mean [21,39].

^d^Legumes included all forms fresh legumes (green peas or green beans), pulses (dry beans, chickpeas, lentils), soya products and legume-based plant-protein products.

^e^Cereals included barley, oat, rice, rye, wheat, starch, other cereals and cereal-based plant-protein products excluding cereal-based drinks.

**Table 5.** Mean daily food consumption and nutrient intake in Finnish women (*n* = 247) aged 65–74 years in the reference diet (FinDiet 2017) and the scenarios in which red and processed meat were partially replaced with legumes or cereals^a^.

	Level	Reference diet		Legume scenario		Cereal scenario		Combination scenario	
		Mean	95% CI	Mean	95% CI	Mean	95% CI	Mean	95% CI
Red and processed meat (g)^b^	70	54.4	48.9–59.9	43.5^c^	40.3–46.6	43.5^c^	40.3–46.6	43.5^c^	40.3–46.6
	30			24.3^c^	23.0–25.5	24.3^c^	23.0–25.5	24.3^c^	23.0–25.5
Legumes (g)^d^	70	9.4	6.3–12.5	19.7^c^	15.2–24.1	9.4	6.3–12.5	14.5	11.1–18.0
	30			37.7^c^	32.2–43.2	9.4	6.3–12.5	23.5^c^	19.7–27.4
Cereals (g)^e^	70	104	97.0–111	104	97.0–111	110	103–117	107	100–114
	30			104	97.0–111	121^c^	113–128	112	105–120
Energy (MJ)	70	6.6	6.4–6.9	6.6	6.3–6.8	6.6	6.4–6.9	6.6	6.4–6.8
	30			6.5	6.2–6.7	6.6	6.4–6.9	6.6	6.3–6.8
Protein (E%)	70	17.3	16.7–18.0	17.2	16.6–17.9	17.0	16.4–17.7	17.1	16.5–17.8
	30			16.8	16.2–17.5	16.3	15.7–17.0	16.6	15.9–17.2
Protein (g)	70	65.7	63.2–68.2	64.7	62.3–67.1	64.5	62.1–66.9	64.6	62.2–67.0
	30			62.4	60.0–64.8	61.9	59.5–64.3	62.1	59.7–64.6
Protein (g/kg BW)	70	0.91	0.87–0.96	0.90	0.86–0.95	0.90	0.85–0.94	0.90	0.85–0.95
	30			0.87	0.82–0.92	0.86	0.82–0.91	0.87	0.82–0.91
Histidine (mg/kg BW)	70	27.4	26.0–28.9	26.7	25.3–28.1	26.6	25.2–28.0	26.7	25.3–28.0
	30			25.1	23.7–26.4	24.9	23.5–26.2	25.0	23.6–26.3
Isoleucine (mg/kg BW)	70	37.4	35.4–39.4	36.7	34.8–38.7	36.5	34.6–38.5	36.6	34.7–38.6
	30			35.3	33.3–37.2	34.8	32.8–36.7	35.0	33.1–37.0
Leucine (mg/kg BW)	70	69.4	65.8–73.1	68.3	64.7–71.9	68.0	64.4–71.7	68.2	64.5–71.8
	30			65.8	62.1–69.4	65.0	61.4–68.6	65.4	61.8–69.0
Lysine (mg/kg BW)	70	60.2	56.9–63.4	58.7	55.6–61.9	58.6	55.4–61.7	58.7	55.5–61.8
	30			55.7	52.5–58.8	55.2	52.1–58.4	55.4	52.3–58.6
Methionine + cysteine (mg/kg BW)	70	32.3	30.7–33.9	31.6	29.9–33.2	31.7	30.1–33.3	31.6	30.0–33.2
	30			30.0	28.4–31.6	30.3	28.7–31.9	30.2	28.6–31.8
Phenylalanine + tyrosine (mg/kg BW)	70	71.8	68.0–75.7	70.9	67.1–74.7	70.8	67.0–74.6	70.8	67.0–74.6
	30			68.8	65.0–72.6	68.5	64.7–72.3	68.6	64.9–72.4
Threonine (mg/kg BW)	70	33.0	31.3–34.8	32.5	30.7–34.2	32.3	30.5–34.0	32.4	30.6–34.1
	30			31.2	29.5–32.9	30.7	28.9–32.4	30.9	29.2–32.7
Tryptophan (mg/kg BW)	70	12.0	11.4–12.6	11.8	11.2–12.4	11.8	11.2–12.4	11.8	11.2–12.4
	30			11.3	10.7–12.0	11.3	10.7–11.9	11.3	10.7–11.9
Valine (mg/kg BW)	70	45.1	42.7–47.5	44.4	42.1–46.8	44.3	42.0–46.6	44.4	42.0–46.7
	30			42.9	40.6–45.3	42.6	40.2–44.9	42.8	40.4–45.1
Total indispensable amino acids (mg/kg BW)	70	389	368–409	382	362–402	381	361–401	381	361–401
	30			366	346–386	363	343–383	365	345–385

^a^In the replacement scenarios, the amount of red and processed meat exceeding 70 g/day or 30 g/day was replaced with the same amount (g) of legume, cereal or the combination (legume 50%, cereal 50%) aggregate while keeping the consumption of other foods unchanged.

^b^Red meat included beef, pork, lamb, game and offal and processed meat included sausages and cold cuts.

^c^Different from the reference diet based on non-overlapping 95% confidence interval (CI) around the mean [21,39].

^d^Legumes included all forms fresh legumes (green peas or green beans), pulses (dry beans, chickpeas, lentils), soya products and legume-based plant-protein products.

^e^Cereals included barley, oat, rice, rye, wheat, starch, other cereals and cereal-based plant-protein products excluding cereal-based drinks.

In the replacement scenarios, the mean daily protein intakes ranged from 15 E% (0.80 g/kg BW) in men aged 65–74 years in the cereal scenario at the 30-g level to 18 E% (1.1 g/kg BW) in men aged 18–64 years in the legume scenario at the 70-g level (Tables 2–5).

### Protein and indispensable amino acid intakes against estimated average requirements

**Figure 1.**
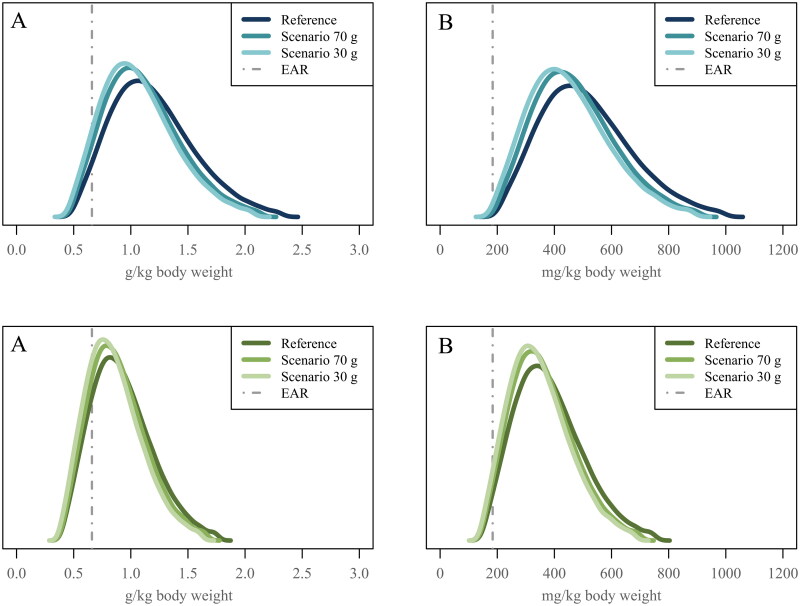
Usual intake distributions for protein (A) and total indispensable amino acids (B) in Finnish men aged 18–64 years (upper panel) and Finnish men aged 65–74 years (lower panel) in the National FinDiet 2017 Survey (reference) and two scenarios in which the consumption of red and processed meat is limited to no more than 70 g/day (scenario 70 g, corresponding to the Finnish nutrition recommendation maximum 500 g/week [6]) or to no more than 30 g/day (scenario 30 g, corresponding to the EAT-Lancet recommendation maximum 200 g/week [4]). In the scenarios, for each subject, the amount exceeding the limit was replaced by the same amount of legumes.

In men aged 18–64 years, the proportion below the EAR for protein intake (g/kg BW) was 5% in the reference diet (Figure 1, Table S2). In the legume and cereal scenarios, the proportions were 7% for both at the 70-g level, and 9% and 10% at the 30-g level, respectively. In men aged 65–74 years, the proportion was 17% in the reference diet (Figure 1, Table S3). In the legume and cereal scenarios, the proportions were 20% and 21% at the 70-g level and 24% and 25% at the 30-g level, respectively.

**Figure 2.**
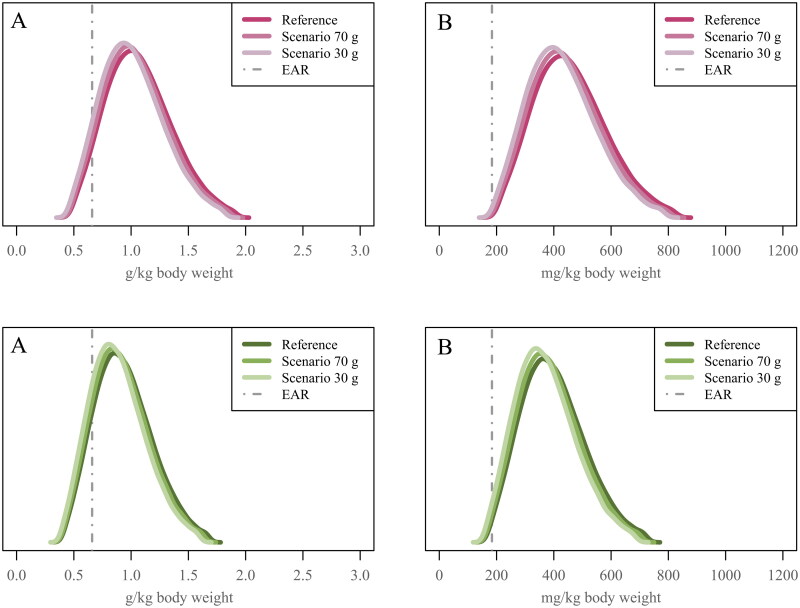
Usual intake distributions for protein (A) and total indispensable amino acids (B) in Finnish women aged 18–64 years (upper panel) and Finnish women aged 65–74 years (lower panel) in the National FinDiet 2017 Survey (reference) and two scenarios in which the consumption of red and processed meat is limited to no more than 70 g/day (scenario 70 g, corresponding to the Finnish nutrition recommendation maximum 500 g/week [6]) or to no more than 30 g/day (scenario 30 g, corresponding to the EAT-Lancet recommendation maximum 200 g/week [4]). In the scenarios, for each subject, the amount exceeding the limit was replaced by the same amount of legumes.

In women aged 18–64 years, the proportion below the EAR for protein intake (g/kg BW) was 7% in the reference diet (Figure 2, Table S4). In the legume and cereal scenarios, the proportions were 8% for both at the 70-g level, and 10% for both at the 30-g level. In women aged 65–74 years, the proportion was 14% in the reference diet (Figure 2, Table S5). In the legume and cereal scenarios, the proportions were 16% and 17% at the 70-g level, and 19% and 20% at the 30-g level, respectively.

For indispensable amino acid intakes (mg/kg BW), the proportions below the EAR were highest for leucine and valine both in the reference diet and the scenarios. In men aged 18-64 years, the proportions below EAR for leucine and valine intake were 1–2% in the reference diet (Table S2). In the legume and cereal scenarios at both levels, the proportions were 2–3%. In men aged 65–74 years, the proportions below EAR for leucine and valine intake were 8% in the reference diet (Table S3). In the legume and cereal scenarios, the proportions were 8–10% at the 70-g level and 10–12% at the 30-g level. For total indispensable amino acids, the proportions below the EAR were ≤1% in men of working age and <5% in older men in the reference diet and the scenarios (Figure 1, Table S2 and S3).

In women aged 18–64 years, the proportions below EAR for leucine and valine intake were 2% in the reference diet (Table S4). In the legume and cereal scenarios at both levels, the proportions were 2–3%. In women aged 65–74 years, the proportions below EAR for leucine and valine intake were 4–5% in the reference diet (Table S5). In the legume and cereal scenarios, the proportions were 5–6% at the 70-g level and 6–7% at the 30-g level. For total indispensable amino acids, the proportions below the EAR were <1% in women of working age and ≤2% in older women in the reference diet and the scenarios (Figure 2, Table S4 and S5).

## Discussion

In this study, we investigated the impacts of partial replacement of red and processed meat with legumes or cereals on the protein and indispensable amino acid intakes in the Finnish adult population. In the replacement scenarios, the amount of red and processed meat exceeding the Finnish nutrition recommendation or the EAT-Lancet recommendation was replaced with the same amounts of legumes, cereals or their combination while keeping the consumption of other foods unchanged. We found that the protein and indispensable amino acid intakes decreased in the replacement scenarios depending on gender and age, and the highest percentages below the EAR were observed in elderly population.

Our current results are in line with previous dietary modelling studies where animal-based foods were replaced with plant-based foods [21–29]. There was a decline in protein intake when meat and dairy foods were partially (30%) or fully (100%) replaced with plant-based alternatives in the Dutch adult population (*n* = 2102) [24]. Similarly, the protein intake decreased when currently consumed plant foods were doubled, and commensurate amounts of animal products decreased in the US populations aged ≥2 years (*n* = 17,387) [21], and ≥51 years (*n* = 5389) [22]. However, these studies also showed that doubling currently consumed protein-rich plant-based foods (i.e. legumes, nuts, seeds, soy) with a commensurate decrease in animal products had no effect on protein intake because these foods were consumed in very low quantities in the baseline diet. Additionally, in a Canadian population aged ≥1 year (*n* = 20,487), there was a decrease in protein intake when currently consumed plant-based meat alternatives (i.e. legumes, seeds and nuts) were doubled and red and processed meat reduced by half [23]. In a Swedish population aged 18–80 years (*n* = 1797), protein intake remained on average within the recommended range when meat was reduced by 50% and replaced with legumes [28]. In our study, only red and processed meat was replaced with plant-based foods, and the consumption of other animal-based foods such as milk products and poultry was kept unchanged. Therefore, the changes in protein and amino acid intakes were relatively moderate in the replacement scenarios of this study.

In this study, also the proportion of the population not meeting EAR for protein and indispensable amino acid intakes were calculated. In men of working age, the percentages below EAR for protein were 5% in the reference diet and 7-10% in the scenarios. In older men, the percentages were notably higher being 17% in the reference diet and 20-25% in the scenarios. In women, the results resembled those of men. Also in previous studies, the transition towards plant-based diets tended to increase the proportion of the population not meeting the EAR for protein [21, 22, 24–26]. Houchins et al. found that when currently consumed plant foods were doubled, and commensurate amounts of animal products decreased in the US population, the percentages below the EAR in protein intake were 13% in men and 23% in women aged 51–70 years (reference 2–6%), and 27% in men and 33% in women aged ≥71 years (reference 7–10%) [22]. Thus, the percentages below the EAR for protein intake were primarily highest in older age groups, which was also observed in our study. However, Houchins et al. observed the highest percentages below the EAR for protein intake in elderly women, whereas we found the highest percentages in elderly men. Gender differences between studies may be explained by differences in the study design or differences in the dietary habits of the populations. Nevertheless, these studies revealed that a significant proportion of elderly men and women had protein intake below the EAR which needs to be considered particularly in transition towards more plant-based diets.

Our study is among the first to report the impacts of the partial replacement of red and processed meat with plant-based foods on amino acid intake in a Nordic population. We found that transition towards more plant-based diets decreased indispensable amino acid intakes depending on gender and age. However, the changes were relatively small especially when red and processed meat was replaced with legumes. These findings are in line with previous modelling studies, where indispensable amino acid intakes and their adequacy were investigated [26,27]. A study in a French population aged 18–65 years (*n* = 1678) suggested that an increase in the plant:animal protein ratio might lead to inadequate protein and lysine intakes if keeping with the current mainly cereal-derived plant-protein intake pattern [26]. The introduction of a higher proportion of legumes, nuts and seeds when substituting for animal protein led to an adequate protein and lysine intake with higher plant:animal protein ratio in the diet. Another study in French population aged 18–74 years (*n* = 2028) found that indispensable amino acid requirements remained covered when the quantity of pulses was raised to the recommended level (i.e. 57 g/day), in replacement of an equivalent portion of meat [27]. Thus, favouring protein-rich plant foods such as legumes may secure adequate protein and amino acid intakes also in more plant-based diets.

The protein quantity and quality differ in legumes and cereals, which was also observed in the protein and indispensable amino acid intakes in our modelling study. The protein and indispensable amino acid contents are somewhat higher in legumes than in cereals [35]. Lysine is present in lower proportions in cereals, and the sulphur-containing amino acids (methionine and cysteine) are proportionally lower in legumes [41]. Dietary proteins of animal origin (meat, fish, milk and eggs) or a combination of plant protein from legumes and cereals will give a good distribution of indispensable amino acids [5]. Mariotti and Gardner concluded in a review that a transition towards 100% plant protein leads to sufficient intake of protein, including amino acids such as lysine, when replacing animal protein with a mix of protein-rich plant foods, namely legumes, nuts and seeds [41]. Similarly, Salomé et al. concluded that plant-based substitutes that include legumes appear to be more nutritionally adequate to substitute animal products than other substitutes [25]. Overall, sufficient protein intake seems to secure adequate intake of indispensable amino acids also in more plant-based diets [26,41].

In the adult population of the Nordic and Baltic countries in 2007–2020, the protein contribution ranged between 15 and 19 energy percent (E%) [8]. In general, based on the FinDiet 2017 Survey [10], the current protein intake of approximately 18 E% in the Finnish adult population is closer to the upper limit of the recommendation (10–20 E%) [5, 6]. Across the gender and age groups of this study, the lowest mean daily protein intake was 15 E%, and it was observed in elderly men in the cereal scenario at the 30-g level. Thus, the mean daily protein intake (E%) remained sufficient also when substantial amounts of red and processed meat was replaced with legumes or cereals.

A notable proportion of especially elderly men and women fall below EAR in protein intake (0.66 g/kg body weight) in this study. This may be partly explained by the excess body weight of the study population. Both working age and elderly men and women were overweight (BMI > 25 kg/m^2^), and the BMI was higher in elderly compared to working age population. Overweight in the study population may have resulted in slightly higher proportions below EAR in protein intake. However, changes in protein and indispensable amino acid intakes against EAR were relatively small between the reference diet and the scenarios.

Strengths of this study are the comprehensive food consumption data collected in the National FinDiet 2017 Survey [10]. Also, non-participation bias correction using survey weights for the food consumption data improves the representativeness of the results to the Finnish adult population [33]. In addition, the use of national food composition database Fineli® with recently updated and comprehensive amino acid composition data [35–38] increases the reliability of the data. Further, we used the SPADE method [40] to estimate the usual intake distributions and to assess the population shares that fall below EAR.

This modelling study also has some limitations. We did not consider other important sources of animal protein, such as milk and poultry, whose consumption is also likely to decline as part of the shift towards more plant-based diets. Furthermore, any misreporting of food intake, such as energy underreporting, was not considered in this study, which may have led to an underestimation of protein and amino acid intakes. Plant-based foods not only contain less protein and amino acids compared to animal-based foods, but also the digestibility of plant protein is lower and varies between plant foods [16]. In this study, we focused on changes in estimated protein and amino acid intakes when red and processed meat are partially replaced by legumes or cereals, and the digestibility of proteins was not considered. This study covered the Finnish adult population aged 18–64 and 65–74 years and did not take into account other age groups (e.g. children) and countries. In future studies, the above-mentioned aspects should be considered to further evaluate and tackle possible challenges of the transition towards more plant-based and sustainable diets.

## Conclusions

Modelling of partial replacement of red and processed meat with legumes or cereals decreased the protein and indispensable amino acid intakes depending on gender and age in the Finnish adult population. The mean daily protein intake of total energy intake remained sufficient also when substantial amounts of red and processed meat were replaced with legumes or cereals. However, in more plant-based diets, a significant proportion of elderly men and women had protein intakes below the EAR, which warrants attention and more research. Overall, our results suggest that legumes and cereals comprise a healthy and sustainable alternative to replace red and processed meat as a source of protein and amino acids in the diet of the adult population. These findings may promote public health and nutrition policy efforts to support the health of humans and the planet.

## Supplementary Material

Supplemental Material

## Data Availability

The data of the Finnish Institute for Health and Welfare (THL) are openly available to other researchers for research purposes on a contractual basis.
