# Supplementary material for: The impacts of partial replacement of red and processed meat with legumes or cereals on protein and amino acid intakes: a modelling study in the Finnish adult population
Source: Ann Med. 2023 Nov 17;55(2):2281661. doi: 10.1080/07853890.2023.2281661 (PMC10732208; doi:10.1080/07853890.2023.2281661)
Supplement: Supplemental Material [file IANN_A_2281661_SM6001.docx]

Table S1. The energy, protein, indispensable amino acid, fat and carbohydrate composition of the legume aggregate, cereal aggregate, and the combination of the aggregates (legume 50%, cereal 50%) per 100 g.

|  | Legume aggregate | Cereal aggregate | Combination aggregate |
| --- | --- | --- | --- |
| Energy (kJ) | 438 | 893 | 665 |
| Protein (g) | 9.4 | 7.6 | 8.5 |
| Histidine (mg) | 226 | 178 | 202 |
| Isoleucine (mg) | 378 | 253 | 316 |
| Leucine (mg) | 661 | 487 | 574 |
| Lysine (mg) | 575 | 474 | 525 |
| Methionine + Cysteine (mg) | 200 | 272 | 236 |
| Phenylalanine + Tyrosine (mg) | 704 | 635 | 670 |
| Threonine (mg) | 354 | 227 | 291 |
| Tryptophan (mg) | 106 | 88 | 97 |
| Valine (mg) | 439 | 352 | 395 |
| Total indispensable amino acids (mg) | 3645 | 2967 | 3306 |
| Fat (g) | 1.4 | 1.5 | 1.5 |
| Carbohydrate (g) | 11 | 38 | 25 |
| Fibre (g) | 4.2 | 6.8 | 5.5 |

Table S2. Evaluation of population shares (%) below estimated average requirements in Finnish men (n=576) aged 18-64 years based on usual intake estimation in the reference diet (FinDiet 2017), the legume scenario and the cereal scenario.

|  | Level | Estimated average requirement ^a^ | Reference diet  Estimate %<EAR | Legume scenario  Estimate %<EAR | Cereal scenario  Estimate %<EAR |  |
| --- | --- | --- | --- | --- | --- | --- |
| Protein (g/kg BW) | 70 | 0.66 g/kg BW | 5.0 | 7.0 | 7.4 |  |
|  | 30 |  |  | 8.7 | 9.7 |  |
| Histidine (mg/kg BW) | 70 | 10 mg/kg BW | 0.05 | 0.07 | 0.07 |  |
|  | 30 |  |  | 0.12 | 0.14 |  |
| Isoleucine (mg/kg BW) | 70 | 20 mg/kg BW | 1.0 | 1.3 | 1.6 |  |
|  | 30 |  |  | 2.0 | 2.5 |  |
| Leucine (mg/kg BW) | 70 | 39 mg/kg BW | 1.3 | 2.0 | 2.0 |  |
|  | 30 |  |  | 2.7 | 3.2 |  |
| Lysine (mg/kg BW) | 70 | 30 mg/kg BW | 0.67 | 0.99 | 1.1 |  |
|  | 30 |  |  | 1.5 | 1.6 |  |
| Methionine + Cysteine (mg/kg BW) | 70 | 15 mg/kg BW | 0.40 | 0.63 | 0.58 |  |
|  | 30 |  |  | 1.1 | 0.94 |  |
| Phenylalanine + Tyrosine (mg/kg BW) | 70 | 25 mg/kg BW | 0.05 | 0.07 | 0.07 |  |
|  | 30 |  |  | 0.09 | 0.10 |  |
| Threonine (mg/kg BW) | 70 | 15 mg/kg BW | 0.36 | 0.49 | 0.56 |  |
|  | 30 |  |  | 0.75 | 0.99 |  |
| Tryptophan (mg/kg BW) | 70 | 4 mg/kg BW | 0.02 | 0.03 | 0.04 |  |
|  | 30 |  |  | 0.05 | 0.07 |  |
| Valine (mg/kg BW) | 70 | 26 mg/kg BW | 1.5 | 2.3 | 2.5 |  |
|  | 30 |  |  | 3.1 | 3.5 |  |
| Total indispensable amino acids (mg/kg BW) | 70 | 184 mg/kg BW | 0.40 | 0.58 | 0.62 |  |
|  | 30 |  |  | 0.95 | 1.1 |  |

^a^ The EAR values were those estimated by the WHO/FAO/UNU [15].

Table S3. Evaluation of population shares (%) below estimated average requirements in Finnish men (n=204) aged 65-74 years based on usual intake estimation in the reference diet (FinDiet 2017) and the legume scenario and the cereal scenario.

|  | Level | Estimated average requirement ^a^ | Reference diet  Estimate %<EAR | Legume scenario  Estimate %<EAR | Cereal scenario  Estimate %<EAR |  |
| --- | --- | --- | --- | --- | --- | --- |
| Protein (g/kg BW) | 70 | 0.66 g/kg BW | 17.3 | 19.8 | 20.8 |  |
|  | 30 |  |  | 23.6 | 25.4 |  |
| Histidine (mg/kg BW) | 70 | 10 mg/kg BW | 0.28 | 0.36 | 0.38 |  |
|  | 30 |  |  | 0.84 | 0.94 |  |
| Isoleucine (mg/kg BW) | 70 | 20 mg/kg BW | 4.7 | 7.0 | 6.6 |  |
|  | 30 |  |  | 8.1 | 9.7 |  |
| Leucine (mg/kg BW) | 70 | 39 mg/kg BW | 7.5 | 8.2 | 9.9 |  |
|  | 30 |  |  | 10.5 | 11.8 |  |
| Lysine (mg/kg BW) | 70 | 30 mg/kg BW | 3.2 | 4.3 | 4.5 |  |
|  | 30 |  |  | 7.3 | 8.0 |  |
| Methionine + Cysteine (mg/kg BW) | 70 | 15 mg/kg BW | 1.9 | 2.6 | 2.4 |  |
|  | 30 |  |  | 3.9 | 3.3 |  |
| Phenylalanine + Tyrosine (mg/kg BW) | 70 | 25 mg/kg BW | 0.29 | 0.31 | 0.32 |  |
|  | 30 |  |  | 0.44 | 0.48 |  |
| Threonine (mg/kg BW) | 70 | 15 mg/kg BW | 1.8 | 2.3 | 2.6 |  |
|  | 30 |  |  | 2.9 | 3.7 |  |
| Tryptophan (mg/kg BW) | 70 | 4 mg/kg BW | 0.14 | 0.16 | 0.17 |  |
|  | 30 |  |  | 0.25 | 0.29 |  |
| Valine (mg/kg BW) | 70 | 26 mg/kg BW | 8.4 | 9.1 | 9.6 |  |
|  | 30 |  |  | 10.4 | 11.4 |  |
| Total indispensable amino acids (mg/kg BW) | 70 | 184 mg/kg BW | 2.8 | 3.5 | 3.7 |  |
|  | 30 |  |  | 4.2 | 4.7 |  |

^a^ The EAR values were those estimated by the WHO/FAO/UNU [15].

Table S4. Evaluation of population shares (%) below estimated average requirements in Finnish women (n=628) aged 18-64 years based on usual intake estimation in the reference diet (FinDiet 2017) and the legume scenario and the cereal scenario.

|  | Level | Estimated average requirement ^a^ | Reference diet  Estimate %<EAR | Legume scenario  Estimate %<EAR | Cereal scenario  Estimate %<EAR |  |
| --- | --- | --- | --- | --- | --- | --- |
| Protein (g/kg BW) | 70 | 0.66 g/kg BW | 6.7 | 7.8 | 8.0 |  |
|  | 30 |  |  | 9.5 | 10.1 |  |
| Histidine (mg/kg BW) | 70 | 10 mg/kg BW | 0.07 | 0.09 | 0.10 |  |
|  | 30 |  |  | 0.15 | 0.16 |  |
| Isoleucine (mg/kg BW) | 70 | 20 mg/kg BW | 1.1 | 1.4 | 1.4 |  |
|  | 30 |  |  | 1.8 | 2.1 |  |
| Leucine (mg/kg BW) | 70 | 39 mg/kg BW | 1.6 | 1.9 | 2.0 |  |
|  | 30 |  |  | 2.5 | 2.8 |  |
| Lysine (mg/kg BW) | 70 | 30 mg/kg BW | 0.61 | 0.79 | 0.81 |  |
|  | 30 |  |  | 1.2 | 1.3 |  |
| Methionine + Cysteine (mg/kg BW) | 70 | 15 mg/kg BW | 0.41 | 0.53 | 0.51 |  |
|  | 30 |  |  | 0.81 | 0.73 |  |
| Phenylalanine + Tyrosine (mg/kg BW) | 70 | 25 mg/kg BW | 0.07 | 0.09 | 0.09 |  |
|  | 30 |  |  | 0.11 | 0.11 |  |
| Threonine (mg/kg BW) | 70 | 15 mg/kg BW | 0.34 | 0.42 | 0.45 |  |
|  | 30 |  |  | 0.57 | 0.69 |  |
| Tryptophan (mg/kg BW) | 70 | 4 mg/kg BW | 0.04 | 0.05 | 0.05 |  |
|  | 30 |  |  | 0.07 | 0.08 |  |
| Valine (mg/kg BW) | 70 | 26 mg/kg BW | 1.9 | 2.3 | 2.4 |  |
|  | 30 |  |  | 2.9 | 3.1 |  |
| Total indispensable amino acids (mg/kg BW) | 70 | 184 mg/kg BW | 0.45 | 0.56 | 0.58 |  |
|  | 30 |  |  | 0.78 | 0.85 |  |

^a^ The EAR values were those estimated by the WHO/FAO/UNU [15].

Table S5. Evaluation of population shares (%) below estimated average requirements in Finnish women (n=247) aged 65-74 years based on usual intake estimation in the reference diet (FinDiet 2017) and the legume scenario and the cereal scenario.

|  | Level | Estimated average requirement ^a^ | Reference diet  Estimate %<EAR | Legume scenario  Estimate %<EAR | Cereal scenario  Estimate %<EAR |  |
| --- | --- | --- | --- | --- | --- | --- |
| Protein (g/kg BW) | 70 | 0.66 g/kg BW | 14.4 | 16.1 | 16.5 |  |
|  | 30 |  |  | 19.0 | 20.0 |  |
| Histidine (mg/kg BW) | 70 | 10 mg/kg BW | 0.22 | 0.28 | 0.30 |  |
|  | 30 |  |  | 0.45 | 0.49 |  |
| Isoleucine (mg/kg BW) | 70 | 20 mg/kg BW | 3.1 | 3.7 | 3.9 |  |
|  | 30 |  |  | 4.8 | 5.4 |  |
| Leucine (mg/kg BW) | 70 | 39 mg/kg BW | 4.1 | 4.9 | 5.1 |  |
|  | 30 |  |  | 6.1 | 6.7 |  |
| Lysine (mg/kg BW) | 70 | 30 mg/kg BW | 1.7 | 2.2 | 2.3 |  |
|  | 30 |  |  | 3.2 | 3.4 |  |
| Methionine + Cysteine (mg/kg BW) | 70 | 15 mg/kg BW | 1.2 | 1.6 | 1.5 |  |
|  | 30 |  |  | 2.2 | 2.0 |  |
| Phenylalanine + Tyrosine (mg/kg BW) | 70 | 25 mg/kg BW | 0.22 | 0.25 | 0.26 |  |
|  | 30 |  |  | 0.34 | 0.35 |  |
| Threonine (mg/kg BW) | 70 | 15 mg/kg BW | 1.1 | 1.3 | 1.4 |  |
|  | 30 |  |  | 1.7 | 2.0 |  |
| Tryptophan (mg/kg BW) | 70 | 4 mg/kg BW | 0.13 | 0.17 | 0.17 |  |
|  | 30 |  |  | 0.23 | 0.25 |  |
| Valine (mg/kg BW) | 70 | 26 mg/kg BW | 4.9 | 5.6 | 5.8 |  |
|  | 30 |  |  | 6.9 | 7.3 |  |
| Total indispensable amino acids (mg/kg BW) | 70 | 184 mg/kg BW | 1.4 | 1.7 | 1.7 |  |
|  | 30 |  |  | 2.2 | 2.3 |  |

^a^ The EAR values were those estimated by the WHO/FAO/UNU [15].
